# Pulmonary arterial catheter vs. prediction index software in patients undergoing orthotopic liver transplantation: “We cannot lump together everything”

**DOI:** 10.1016/j.bjane.2025.844605

**Published:** 2025-03-03

**Authors:** Luigi Vetrugno

**Affiliations:** University of Chieti-Pescara, Department of Medical, Oral and Biotechnological Sciences, Chieti, Italy

*Dear Editor,*

We read with great interest the article by Cywinski JB et al. regarding the agreement between cardiac output and systemic vascular resistance measured with HPI software (HemoSphere with Acumen IQ sensor platform ‒ Edwards Lifesciences Corp. One Edwards Way, Irvine, CA 92614) and the Pulmonary Artery Catheter (PAC) during Liver Transplantation (LT).^1^

The authors compared Cardiac Output (CO) and Systemic Vascular Resistance (SVR) through the analysis of arterial waveform time, amplitude, area, segment slopes, and complexity to predict arterial hypotension, defined as a Mean Arterial Pressure (MAP) of less than 65 mmHg lasting at least one minute.

Not surprisingly, the aggregated results from this study of 23 adult liver transplantation patients, which included 125 pairs of CO measurements and 122 pairs of SVR measurements between PAC and HPI, were not interchangeable when using a relative difference of less than 20% or a clinically acceptable level of agreement defined as ±1L.min^-1^ for CO and ±200 dynes.s.cm^-5^ for SVR.

The Bland-Altman limits of agreement analysis revealed a bias of 1.96 L.min^-1^ (SD=2.74 L.min^-1^) for CO, with a 95% limit of agreement of -3.42 L.min^-1^ (95% CI: -5.00, -2.34) and 7.34 L.min^-1^ (95% CI: 6.26, 8.92). For SVR, the bias was -93 dynes.s.cm^-5^ (SD=241 dynes.s.cm^-5^), with 95% limits of agreement of -565 dynes.s.cm^-5^ (95% CI: -729, -456) and 379 dynes.s.cm^-5^ (95% CI: 270, 543).

Conversely, the authors discovered that of over 1860 HPI alerts (HPI ≥85), 618 events were predicted by HPI alerts, with 614 confirmed as “true alerts” and a median time from HPI alert to hypotension of 3.3 minutes. This indicates that the software demonstrated high sensitivity but low specificity for predicting hypotension.

We agree with the authors that the hemodynamics in liver transplant patients are complex. This complexity arises from several factors, including the potential presence of cirrhotic cardiomyopathy, abnormalities in vascular tone, and the risk of acute right or left cardiac failure during critical phases of the procedure, such as declamping and reperfusion of the new graft.^2^ While the Pulmonary Artery Catheter (PAC) can be useful for monitoring, it is also the most invasive hemodynamic tool. As a result, some anesthesiologists prefer to use it only in severe cases with a high Model for End-Stage Liver Disease (MELD) score. It is important to note that higher MELD scores are associated with an increased risk of bleeding and hemodynamic instability, necessitating careful monitoring and management.^3^^,^^4^ However, Transesophageal Echocardiography (TEE) is another option for monitoring rapid hemodynamic changes during surgery, albeit it does not allow for continuous cardiac output measurement.^5^^,^^6^ For patients with lower MELD scores, TEE and HPI may be preferable for continuous cardiac output monitoring and hypotension prediction.

The study by Cywinski JB et al. merits considerable attention, and we wish to contribute to this discussion as follows: first, while the PAC catheter is widely accepted as the clinical gold standard, it is important to note that the percentage error of PAC compared to the true gold standard, an aortic flow probe in CO measurement, has been shown to exceed 40%.^1^ Recent suggestions by Payet et al. indicate that a percentage error of 45% should be accepted in clinical practice, rather than the 20% proposed by the authors.^7^ Second, observing the Bland-Altman analysis results, it appears that the greatest variability in measurement accuracy occurs at higher CO values, aligning with previously reported data in the literature.

Finally, we invite the authors to refrain from combining all data and instead report the results phase by phase for a clearer understanding of when HPI monitoring is most beneficial. Specifically, we are curious whether the HPI software leads to better predictions of true hypotensive events during the hepatectomy phase using the modified “piggyback” technique compared to the anhepatic phase or the reperfusion phase. We suggest dividing the data analysis into four phases: Basal (T1), Anhepatic (T2), Reperfusion (T3), and End (T4). Previous studies suggest that less invasive devices may perform differently across these phases, and ejection fraction may impact CO and potentially HPI accuracy.^8^^,^^9^

We also wonder if many false HPI alerts were concentrated during the reperfusion phase. Additionally, we kindly request the authors to discuss any modifications induced by the recipient's MELD status, as the data from your population of LT patients showed a MELD score of 19.4±8.2, which is relatively high. In conclusion, as technology progresses, anesthesia monitoring should prioritize less invasiveness, but it is crucial to define which surgical phase this is most applicable.

## Authors’ contributions

Luigi Vetrugno: Conceptualized, write and prepare the manuscript.

## Funding

None.

## Conflicts of interest

The authors declare no conflicts of interest.
